# Yearly Height Gain Is Dependent on the Truly Received Dose of Growth Hormone and the Duration of Periods of Poor Adherence: Practical Lessons From the French Easypod™ Connect Multicenter Observational Study

**DOI:** 10.3389/fendo.2021.790169

**Published:** 2022-01-20

**Authors:** Régis Coutant, Marc Nicolino, Benoit Cammas, Valérie de Buyst, Maïthé Tauber, Jean-François Hamel

**Affiliations:** ^1^ Pediatric Endocrinology and Diabetology Department, Angers University Medical Center, Angers, France; ^2^ Pediatric Endocrinology, Diabetology and Metabolism Department, Lyon Women’s and Children’s Hospital, Lyon, France; ^3^ Pediatric Endocrinology Department, Bordeaux Nord Aquitaine Clinic, Bordeaux, France; ^4^ Medical Affairs Department, Merck Serono SAS, an Affiliate of Merck KGaA, Lyon, France; ^5^ Endocrine, Obesity Bone Diseases Gynecology and Genetics Unit, Children’s Hospital, Toulouse University Medical Center, Toulouse, France, and Toulouse Institute for Infectious and Inflammatory Diseases, INSERM UMR1291 - CNRS UMR5051, Toulouse III University, Toulouse, France; ^6^ Biostatistics and Methodology Department, Angers University Medical Center, Angers, France, and ESTER Group, IRSET INSERM UMR 1085, Angers University, Angers, France

**Keywords:** recombinant human growth hormone, children, height gain, adherence, electronic device, growth hormone deficiency, small for gestational age

## Abstract

**Objective:**

To study the impact of the true mean daily dose and the true mean number of injections per week on the yearly height gain in short children treated with recombinant human growth hormone (rhGH).

**Design and Methods:**

220 children from the French Easypod™ Connect Observational Study (ECOS) used the Easypod™ electronic device to record rhGH injections. The mean daily rhGH dose (the sum of the doses truly received divided by the number of days) and mean number of injections per week (the number of injections truly performed divided by the number of weeks) were calculated. Linear mixed models were used to study the impact of short (3-month) and long (1-year) variations in rhGH administration on the yearly height change [as a standard deviation score (SDS)], with time on treatment as a covariate. For each patient, several periods of 3 or 12 months were considered and designated as poorly adherence or fully adherence. We studied the impact of each of period on the height change.

**Results:**

At treatment initiation, the mean ± SD age was 9.8 ± 3.7 years (females: 47%, prepubertal: 86%) and the mean height was -2.28 ± 0.92 SDS. The mean treatment duration was 3.2 ± 1.1 years (685.2 patient years). 122 patients were GH-deficient, 79 were small for gestational age, and 19 had Turner syndrome. When treatment was computed over 12-month periods, receiving a mean daily dose <0.03 mg/kg.d was associated with a 20% lower mean yearly height gain SDS when<3 injections/week were received (vs.>5 injections/week), whereas maintaining a mean daily dose >0.03 mg/kg.d with<3 injections/week was not associated with a lower yearly height gain SDS (vs.>5 injections/week). For 3-month periods, changes in the daily rhGH dose or the number of injections per week over such short period did not influence the yearly height gain SDS.

**Conclusion:**

The 12-month treatment model showed that when poor adherence leads to a low true daily GH dose, the yearly height gain is low. The 3-month treatment model showed that poor adherence for short periods (<3 months) had no impact on the height SDS.

## 1 Introduction

Several height prediction models in patients treated with recombinant human growth hormone (rhGH) have been published over the last years (1–6). The models were derived from multiple regression analyses of retrospective data from large national or international registries and were intended to guide treatments with rhGH. Most of the models were designed to predict the height gain (expressed as a standard deviation score (SDS)) in various conditions with short stature, such as growth hormone deficiency (GHD), small for gestational age (SGA), and Turner syndrome (TS) (2, 7). Most models considered the height gain over the first year, the second year, the third year, and then later on, and sought to explain these endpoints with regard to several covariates. The application of these models revealed factors that influence the growth response to rhGH treatment and provided insight into how rhGH dose regimens could be personalized over the first years of treatment (8). In patients of short stature (regardless of the underlying cause), the same auxological variables were generally found to be significant in all the first-year models: age at treatment initiation, birth weight or birth length SDS, weight and/or height SDS at treatment initiation, midparental height SDS, prescribed rhGH dose, and the number of injections per week) (2, 7). Strategies used to personalize the GH dosage include (i) prediction model-based dosing, based on estimated responsiveness whereby the patient’s baseline auxological and biochemical characteristics determine the starting GH dose; (ii) auxology-based dosing, in which the height-based GH dose is increased if the growth response is lower than expected, and (iii) insulin-like-growth factor (IGF-I)-based dosing, where the GH dose is titrated to achieve a desired IGF-I (SDS) level (9). Surprisingly, the impact of the rhGH dose is attenuated or null after the first few years (2, 7, 10–14).

All the models have one major inherent limitation: the rhGH dose level and injection frequency prescribed by the physician are not always those truly received by the child. It was long thought that adherence was best evaluated by counting the number of rhGH vials requested or returned by the child’s family (15). The recent use of electronic devices (such as the Easypod™) to record injections actually performed has enabled reliable evaluations of adherence to treatment. In fact, various studies have found that adherence is very high during the first years of treatment but then decreases over time (16, 17). Overall, the research suggests that predictive models based on the prescribed rhGH treatment are not accurate after the first few years of treatment. Poor adherence is known to be related to a poor growth response to rhGH treatment; however, the relationship was not studied beyond the first 2 years of treatment, when adherence truly decreases - even when the latter was measured accurately with the Easypod™ electronic device (15–19).

In the present study, we used the Easypod™ to assess the real-life effect of rhGH treatment on the growth outcome in 220 children enrolled in the French Easypod™ Connect Observational Study (ECOS) (16, 19). However, we used linear mixed models (rather than standard linear regression models) to evaluate the impact of treatment adherence on height SDS. Mixed models are appropriate for repeated measurements and for analyzing the effect of variations in explanatory covariates on the endpoint (the height SDS, in the present study). Rather than studying the endpoint at different time points (1 or 2 years), we considered the duration of treatment as a covariate. The rhGH treatment was described as the mean daily dose (the sum of the truly received doses of treatment divided by the number of days in the period in question) and mean number of injections per week (the number of injections truly received divided by the number of weeks in the period in question), and the impact of a change in treatment was only considered during the period when it occurred. We believe that linear mixed models are very suitable for detecting the impact of treatment variations (including periods with poor adherence) on the yearly height change. In contrast, linear mixed models might not be ideal for studying the 12-month or 2-year height change because they smooth the first years’ annual height gain, which is usually greater than gains in later years (2, 7, 9). We analyzed the effect of short-term (3-month) and long-term (12-month) variations in rhGH administration on the height SDS in two linear mixed models. Each patient contributed to several 3- or 12-month periods (depending on the unit of time chosen), which made it possible to (i) isolate periods when the patient was poorly adherent and other periods when the same patient was fully adherent, and thus (ii) study the impact of each of these periods on the concurrent height change. A large variation in treatment over a short period – treatment cessation during a one-month-vacation, for example – will have a significant impact on the mean daily dose and number of injections per week when computed over a 3-month period but might have a smaller impact when computed over a 12-month period. In contrast, variations in rhGH administration lasting several months will have an impact on treatment computed over a 12-month period.

## 2 Materials And Methods

### 2.1 Patients and Study Design

We retrospectively analyzed data from 220 participants in the French ECOS study. ECOS is a 5-year, phase IV open-label study that ran between November 2010 and February 2016 in 24 countries, with the objective of describing “real-world” rhGH treatment in short children who were using the Easypod™ electronic drug delivery device (16, 19). Thirty pediatric endocrinology departments from across France participated in the French part of the ECOS study and included patients between January 2011 and December 2015. Patients were aged 2 to 16 years, and none had growth plate fusion. Eligible patients had a baseline visit and then two to four study visits per year, depending on local routine clinical practice. The duration of follow-up ranged from a minimum of 6 months to a maximum of 5 years. All diagnoses and treatment decisions were at the discretion of the investigating physician, in line with standard practice in endocrinology. The study was conducted in accordance with the principles of the Declaration of Helsinki, good clinical practice (ICH-GCP E6) guidelines, and applicable national legal and regulatory requirements. Written informed consent was obtained from patients (or their parent/guardian) prior to study enrolment. In line with the French legislation on retrospective studies of routine clinical practice, the present study was approved by the French National Consultative Committee on Information Processing in Medical Research (*Comité Consultatif sur le Traitement de l’Information en Matiere de Recherche dans le domaine de la Santé*, Paris, France; reference: DGRO CCTIRS MG/CP 10.565). Treatment data (the daily rhGH dose and the frequency of injections performed by each patient) were recorded and collected *via* the Easypod™ device. The device has a skin sensor that should prevent injections into a plant or a toy but does not necessarily prevent injections into a pet, for example. Furthermore, it is recommended that injections be performed under parental supervision, although there is no way to ensure that this actually happens.

Baseline and outcome measures were obtained by physician data entry into clinical report forms.

### 2.2 Statistical Analyses

Continuous variables were expressed as the mean ± standard deviation (SD) and compared using the Kruskal-Wallis test. Categorical variables were expressed as the frequency (percentage) and compared using Fisher’s exact test.

Each child’s height was expressed as an SDS every 3 months, using the French Sempé reference growth curve (20). The height data were analyzed in linear mixed models that took account of repeated measurements over time in the same patient. Since most of the patients were seen every 6 months, linear interpolations were used to complete 3-month data when the child’s height was missing.

The mean daily dose and the number of rhGH injections per week were computed over two different time units (3-month periods and 12-month periods), and so two linear mixed models were used to estimate the impact of the considered period on the children’s height SDS.

The two models included several covariates:

• the midparental height SDS (21).
• the indication for rhGH treatment: isolated idiopathic GHD, complex GHD (and other pituitary hormone deficiencies, if applicable), SGA, and TS.
• the age at rhGH treatment onset (considered as a continuous covariate).
• pubertal status (considered as a time-dependent covariate, meaning that the impact of puberty was considered only for the follow-up period when the child had entered puberty).
• the effect of time (expressed in years) on the height SDS, referred to hereafter as the “time effect”. In the model, the time effect corresponds to the time on rhGH treatment. Even in the 3-month model, the time effect was extrapolated to one year in order to compare the results with those of the 12-month model.
• the duration of rhGH treatment (dichotomized as<or>2 years) considered as a time dependent covariate, given that the treatment’s effect on height is known to be greater during the first years (1, 2, 5–7).
• the mean daily dose of rhGH received (dichotomized as<or>0.03 mg/kg.d), computed as the sum of the truly received doses of treatment in a given period divided by the number of days in that period. This cut-off was chosen because it corresponds to the median recommended dose of rhGH for GHD (0.025 to 0.035 mg/kg.d, according to the prescribing information), and the numbers of time periods respectively below and above 0.03 were equivalent.
• The mean number of rhGH injections per week (categorized as <3 injections/week, 3-to 4 injections/week, and>5 injections/week), computed as the number of injections truly performed in a given period divided by the number of weeks in that period.

Since the number of injections performed per week influenced the truly received mean daily rhGH dose, six treatment categories were studied by combining the two daily rhGH dose categories vs. the three categories for the number of injections per week.

The daily doses recommended in France and Europe for GHD, SGA, and TS are respectively 0.025-0.035 mg/kg.d, 0.035 mg/kg.d (up to 0.050 mg/kg.d), and 0.045-0.05 mg/kg.d (16, 22). For all these indications, performing less than 3 injections/week leads to a calculated mean daily dose<0.030 mg/kg.d. In France, physicians tend to prescribe rhGH as either 6 or 7 doses per week. If 6 doses are given, the mean daily dose is increased accordingly so that the total dose is similar to a regimen with 7 injections/week. In the Easypod™, the prescribed (theoretical) number of injections per week can be set to 6 or 7. In the present study, however, the value of this setting could not be ascertained. We therefore analyzed the number of injections per week and the daily dose, rather than the percentage of the prescribed injections actually administered over a given time period. We defined fair adherence as>5 injections/week and poorly adherence as<3 injections/week.

The interactions between time since treatment onset and all the covariates listed above were considered when studying the impact on the change over time in the children’s height SDS. Only interactions with a p-value below 0.2 were included in the final models.

The linear mixed models included two different random effects: the first at the individual level (illustrating the interindividual variation in height at treatment onset) and the second linked to the time since treatment onset (illustrating the interindividual variation in height change over time). We considered that the random effects did not have a variance-covariance structure and that the residual errors had an exponential structure.

The threshold for statistical significance was set to p<0.05. All the analyses were performed with Stata software (version 13.1, StataCorp LLC, College Station, TX, USA).

## 3 Results

### 3.1 Clinical Characteristics of the Study Population

Two hundred and twenty patients (corresponding to 685.2 patient years) were assessed (**Table 1**). The mean duration of treatment was 3.2 ± 1.1 years (minimum: 1.8 years). The indication for rhGH treatment was GHD for 122 patients, SGA for 79, and TS for 19. At treatment initiation, the mean age was 9.8 ± 3.7 years, the mean height was - 2.28 ± 0.92 SDS, 47% of the participants were female, and 86% were prepubertal. Over the study period as a whole, 106 patients (48%) received a mean daily rhGH dose<0.03 mg/kg.d, 92 (42%) received a mean dose of between 0.03 and 0.045 mg/kg.d, and 22 (10%) received a mean dose >0.045 mg/kg.d. Forty-nine patients with isolated GHD (45%), 51 (64%) patients with SGA and 13 (71%) patients with TS received a mean daily rhGH dose >0.03 mg/kg.d.

**Table 1:** Clinical characteristics of the study participants.

	AllN = 220	Complex GHDN = 13	IsolatedGHD N = 109	SGAN = 79	Turner syndromeN = 19	P
Females/males	47%/53%	46%/54%	37%/63%	49%/51%	100%/0%	<0.0001
Birth weight (Z-score)	-0.91 ± 0.92	-0.53 ± 0.84	-0.62 ± 0.81	-1.35 ± 0.95	-0.91 ± 0.64	<0.0001
Birth length (Z score)	-1.27 ± 0.90	-0.84 ± 0.68	-0.94 ± 0.85	-1.76 ± 0.82	-1.35 ± 0.64	<0.0001
Gestational age (weeks)	38.2 ± 3.0	38.9 ± 2.2	38.8 ± 1.8	37.2 ± 3.9	38.3 ± 2.9	0.095
Midparental height (Z-score)	-0.45 ± 1.7	-0.32 ± 1.60	-0.12 ± 1.59	-0.72 ± 1.78	-1.68 ± 1.19	0.0016
Age at treatment onset (yrs)	9.8 ± 3.7	11.1 ± 4.4	10.5 ± 3.4	9.0 ± 3.4	7.8 ± 4.3	0.0007
Height at T0 (Z score)	-2.28 ± 0.92	-1.71 ± 1.36	-2.05 ± 0.89	-2.66 ± 0.64	-2.51 ± 1.11	0.0001
Weight at T0 (Z-score))	-1.32 ± 1.26	-0.02 ± 1.84	-1.04 ± 1.26	-2.00 ± 0.75	-1.01 ± 1.12	0.3545
BMI at T0 (Z-score)	-0.24 ± 1.50	0.95 ± 1.50	-0.01 ± 1.44	-0.99 ± 1.28	0.59 ± 1.29	0.0001
Puberty (no/yes)	86%/14%	64%/36%	85%/15%	90%/10%	94%/6%	0.0859
Mean rhGH dose (mg/kg.d)*	0.04 ± 0.01	0.03 ± 0.01	0.03 ± 0.01	0.04 ± 0.01	0.04 ± 0.01	0.0003
% of doses in a given range (mg/kg/d)						
<0.03	48%	51%	55%	44%	29%	0.0025
0.03 to 0.045	42%	37%	40%	43%	54%	0.214
>0.45	10%	13%	5%	13%	17%	0.0129

*The mean daily rhGH dose was the dose truly received.

### 3.2 Treatment-Related Factors That Influenced the Height Gain

The 12-month and 3-month models enabled us to assess the effect of a variation in rhGH dose and injection frequency on the yearly height gain SDS over a long period of time and a short period of time, respectively (**Table 2**).

**Table 2:** Linear mixed models for the change in height SDS over 12 months with a time unit of 12 months or 3 months when computing the true mean daily rhGH dose and mean number of injections per week (corresponding to a total of 685.2 patient years, or 2741 patient trimesters).

	12-month model			3-month model		
Height SDS	% of patients.years	Coeff. [95%CI]	p	% of patients.trimester	Coeff. [95%CI]	p value
Constant = mean height at the onset of rhGH treatment		-2.096 [-2.479; -1.713]	<0.001		-2.058 [-2.452; -1.664]	<0.001
Low midparental height (<median for the cohort)		-0.186 [-0.427; 0.056]	0.132		-0.215 [-0.463; 0.034]	0.091
Indication (reference category: isolated GHD)						
complex GHD		0.246 [-0.243; 0.735]	0.324		0.254 [-0.246; 0.753]	0.320
small for gestational age		-0.493 [-0.746; -0.240]	<0.001		-0.497 [-0.757; -0.238]	<0.001
Turner syndrome		-0.260 [-0.700; 0.180]	0.247		-0.273 [-0.723; 0.176]	0.234
Age at initiation of rhGH treatment (+1 year)		0.014 [-0.018; 0.047]	0.389		0.010 [-0.023; 0.044]	0.547
Puberty (yes/no)		0.068 [-0.072; 0.208]	0.343		0.083 [-0.045; 0.210]	0.204
Time effect (+1 year) for rhGH dose>0.03 mg/kg/d and>5 inj/week (ref)	27%	0.372 [0.324; 0.432]	<0.001	45%	0.384 [0.336; 0.444]	<0.001
Change in time effect if rhGH dose>0.03 mg/kg/d and 3 to 4 inj/week	9%	0.012 [-0.036; 0.048]	0.643	4%	0.000 [-0.024; 0.024]	0.82
Change in time effect if rhGH dose>0.03 mg/kg/d and<3 inj/week	7%	-0.012 [-0.060; 0.036]	0.675	2%	-0.024 [-0.048; 0.012]	0.22
Change in time effect if rhGH dose<0.03 mg/kg/j and>5 inj/week	7%	0.048 [0.000; 0.096]	0.072	11%	0.000 [-0.012; 0.024]	0.527
Change in time effect if rhGH dose<0.03 mg/kg/j and 3 to 4 inj/week	13%	0.012 [-0.012; 0.048]	0.357	8%	0.000 [-0.024; 0.012]	0.80
Change in time effect if rhGH dose<0.03 mg/kg/j and<3 inj/week	36%	-0.036 [-0.060; 0.00]	0.044	30%	-0.012 [- 0.024; 0.004]	0.131
Change in time effect if rhGH treatment duration>2 years		-0.024 [-0.060; 0.012]	0.168		-0.024 [-0.036; 0.000]	0.047
Change in time effect if puberty		-0.048 [-0.1008; 0.002]	0.061		-0.048 [-0.096; -0.002]	0.040
Change in time effect if low midparental height		-0.054 [-0.119; 0.012]	0.108		-0.048 [-0.108; 0.024]	0.190

Coeff.: coefficient; inj: injection. There was no significant interaction between time and the cause of short stature. The time effect described the effect of one year of rhGH treatment on the height SDS. Ref = reference category. Even in the 3-month model, the time effect was extrapolated to 12 months for easier comparisons of the two models

#### 3.2.1 The 12-Month Model

The results for the 12-month model showed that the mean height at treatment onset was -2.096 SDS [95% confidence interval (CI): -2.479; -1.713]. Children treated for SGA were significantly smaller at baseline (mean height: - 0.49 SDS [95%CI: -0.75; -0.24], relative to isolated GHD (the reference category).

Membership of the low dose category (<0.03 mg/kg.d) was mostly due to poor adherence (<3 injections/week) (accounting for 64% of the low dose periods), whereas the high dose category (>0.03 mg/kg.d) mostly corresponded to fair adherence (>5 injections/week) (accounting for 63% of the high dose periods).

For all the children, the height SDS increased during the rhGH treatment. The daily dose of rhGH and the weekly number of rhGH injections were significantly associated with the height increase per year (**Table 2**). The mean height increase per year was 0.372 SDS [95%CI: 0.324; 0.432] for the fair adherence/high dose category (the reference category).

**Figure 1:**
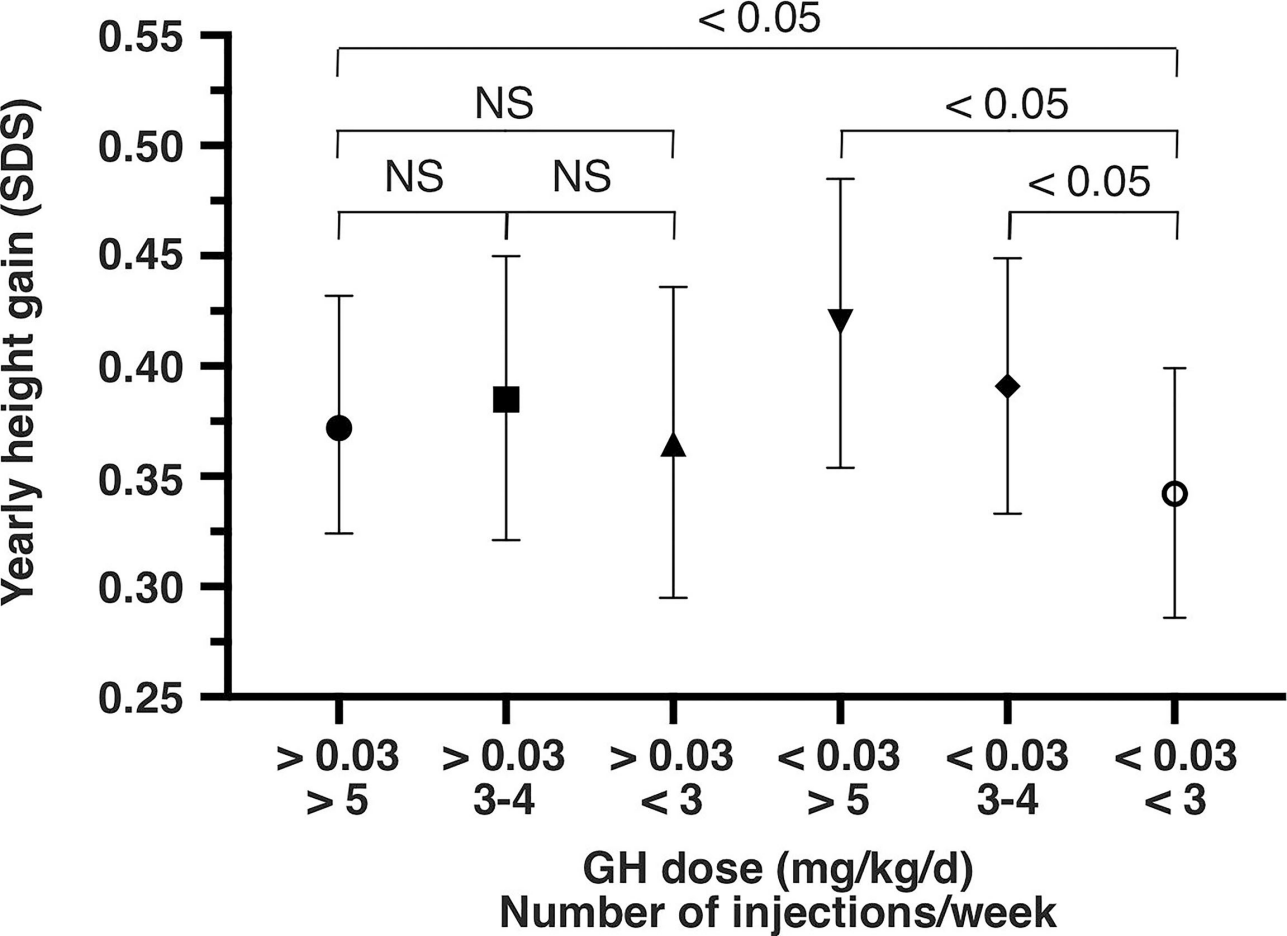
Yearly height gain (SD score and 95% confidence interval) from the 12-month model, as a function of the mean daily rhGH dose (mg/kg/d) and the mean weekly number of injections. SDS, standard deviation score; NS, not significant; GH, growth hormone.

When the mean daily dose of rhGH received was low (<0.03 mg/kg/d)(mean rhGH dose: 0.019 mg/kg/d), the mean height increase per year was 0.342 SDS [95%CI: 0.286 – 0.399] for poor adherence (< 3 injections/week; p<0.05 vs. the reference category). It was 0.391 SDS [95%CI: 0.333–0.449] for intermediate adherence (3 to 4 injections/week; p<0.05 vs. poor adherence), and 0.420 SDS [95%CI: 0.354 – 0.485] for the fair adherence category (>5 injections/week; (p<0.05 vs. poor adherence) (**Figure 1**). In relative terms, the mean yearly height change was 20% lower in the poor adherence/low dose category than in the fair adherence/low dose category.

When the mean daily dose of rhGH received was high (>0.03 mg/kg/d)(mean rhGH dose: 0.039 mg/kg/d), the mean height increase per year was 0.365 SDS [95%CI: 0.295 – 0.436] for poor adherence (NS vs. fair adherence) and 0.385 SDS [95%CI: 0.321 – 0.450] for intermediate adherence (NS vs. fair adherence). The same result (NS vs. fair adherence) was found when the poor and intermediate adherence categories were pooled (i.e. rhGH dose >0.03 mg/kg.d and <5 injections/week).

When the patients entered puberty, the mean height increase per year was 0.05 SDS lower [95%CI: -0.10; 0.00](p = 0.06). When midparental height was low (i.e. below the median midparental height for the cohort), the mean height increase over one year was 0.05 SDS lower [95%CI: -0.12; 0.01](p = 0.11). We did not observe a significant interaction between the time effect and the indication for treatment, suggesting that the cause of short stature did not influence the effect of treatment compliance on the yearly height gain.

#### 3.2.2 The 3-Month Model

The results of the 3-month model (**Table 2**) also showed that mean height at treatment onset was -2.058 SDS [95%CI: -2.452; -1.664]. Children treated for SGA were significantly smaller at treatment onset (mean height SDS - 0.50 [95% confidence interval -0.76; -0.24], relative to isolated GHD (the reference category).

The distribution of the 2741 patient trimesters in each treatment category is described in **Table 2**. Fair adherence accounted for 56% of the 2741 patient trimesters, and poor adherence accounted for 32%. As had been seen in the 12-month model, membership of the low dose category (<0.03 mg/kg.d) was due mostly to poor adherence (accounting for 61% of the low dose periods), and the high dose category (>0.03 mg/kg.d) mostly corresponded to fair adherence (accounting for 88% of the high dose periods).

Fair adherence was observed for 64% of the 3-month periods in the first year, 61% in the second year, and 51% in the third year, evidencing a decrease in adherence with treatment duration. Forty-four percent of the individuals moved from one adherence category to the other during the follow-up.

For all children, the height SDS increased over time. For the reference category (fair adherence/high dose), the mean yearly height change (0.38 SDS [95%CI: 0.34; 0.44]) was close to that observed in the 12-month model. For the other treatment categories, no difference in the height increase per year (compared with the reference category) was observed. As in the 12-month model, the pooling of the smallest categories (intermediate and poor adherence/high dose) gave the same results (NS vs. fair adherence/high dose).

These findings suggest that variations in the rhGH dose and injection frequency over short periods only (3 months, here) did not significantly impact the yearly height gain. After 2 years of treatment, the mean yearly height gain in SDS was significantly lower (-0.024 SDS [95%CI: -0.04; 0.00]; p<0.05). The mean yearly height gain was also lower when the children entered puberty (-0.05 SDS [95%CI: -0.10; -0.00]; p<0.05).

We did not observe a significant interaction between the time effect and the indication for treatment, suggesting that the cause of short stature did not influence the effect of treatment compliance on the yearly height gain.

### 3.3 Safety

The safety data came from the main ECOS France study. Of the 201 patients with safety data, 10 (5.0%) reported a total of 13 serious adverse events (SAEs). Nine of the 10 patients required hospitalization. Two patients had SAEs that led to study discontinuation (1.0%). None of the SAEs was fatal. The SAEs were hemorrhagic diarrhea; viral meningitis, viral myositis, pyrexia and headache, velopharyngeal insufficiency, adenoidal disorder and tympanic membrane disorder, sleep apnea syndrome, respiratory failure, gynecomastia, cancer recurrence, and an anaphylactic reaction. Three patients (1.5%) reported at least one treatment-related SAE: cancer recurrence for the first, adenoidal disorder and tympanic membrane disorder for the second, and gynecomastia for the third.

## 4 Discussion

Our present results highlighted the effect of variations in rhGH administration on the growth outcome in a relatively large French cohort of children using the connected Easypod™ device. Fair adherence (>5 injections/week) was observed for 64% of the periods in the first year, 61% in the second year, and 51% in the third year - evidencing a decrease in adherence with treatment duration. Forty-four percent of the individuals moved from one adherence category to the other during the follow up; this supports our decision to study adherence by considering periods rather than individuals, since adherence status can change over time. When the treatment was computed over 12-month periods, receiving a mean daily dose <0.03 mg/kg.d was associated with a 20% lower mean yearly height gain SDS when <3 injections were performed per week (vs.>5 injections/week), whereas maintaining a mean daily dose >0.03 mg/kg.d despite <3 injections/week was not associated with a lower yearly height gain SDS (vs.>5 injections/week). When the treatment was computed over 3-month periods, the yearly height gain SDS was not influenced by variations in the daily rhGH dose and the number of injections per week. Possible reasons for these apparent paradoxes are discussed below and might shed light on the effect of the interplay between adherence and rhGH sensitivity on the growth outcome.

In France (as in some other countries), physicians typically prescribe either 6 or 7 rhGH injections per week; hence, we considered that the patients performing>5 injections/week were fairly adherent. It is noteworthy that 64% of the low-dose periods were accounted for by poor adherence in the 12-month model (<3 injections/week). We therefore hypothesize that the impact of the number of injections per week on height gain SDS could be explained as follows. Firstly, the low dose group mainly comprised patients with poor adherence (with a low mean daily dose resulting from a low number of injections per week, even when the prescribed dose was>0.03 mg/kg.d), which led to a suboptimal growth outcome. Secondly, adherent patients who were sensitive to rhGH treatment (taking a low mean daily dose but more than 5 times a week) had an adequate growth outcome. In agreement with this hypothesis, several studies have shown that the height increase is higher in patients receiving similar, low weekly doses of rhGH 6 times a week vs. 3 times a week (23, 24).

Conversely, in patients receiving a true mean daily rhGH dose>0.03 mg/kg, the number of injections per week did not influence the height gain in the 12-month model, provided that the daily rhGH dose remained high enough; the mean daily dose in this category was 0.039 mg/kg.d. Patients with fair adherence (>5 injections/week) received a dose close to that prescribed, and this dose range is usually given to patients with some degree of resistance to treatment with rhGH (25). This resistance may be linked to the cause of short stature, and so this phenomenon might be “indication-dependent”. In particular, non-GHD patients may have specific bone structures that are partly responsible for the short stature but are also quite refractory to the effect of rhGH. Indeed, patients with TS or SGA usually receive higher starting rhGH doses than patients with GHD, as they are considered to respond less to treatment. This was true in the present study, and is line with the current treatment guidelines (**Table 1**) (22). However, it is noticeable that some GHD patients also received higher-than-usual doses (**Table 1**). If patients with poor adherence received a mean daily dose of rhGH>0.03 mg/kg.d, the physician must have prescribed even higher doses of rhGH at some point - probably as a result of a poor response to treatment. Although a poorly adherent patient will receive only a portion of this adjusted prescribed dose, it might nevertheless be enough to achieve a mean daily dose of rhGH>0.03 mg/kg.d and thus a good growth outcome. In contrast, not adjusting the prescribed rhGH dose in non-adherent patients would cause the calculated daily dose of rhGH to fall below 0.03 mg/kg.d – making a suboptimal growth outcome more likely. Our results are reassuring with regard to the treatment of children and adolescents in whom levels of adherence are often suboptimal; the dose increase prescribed by the physician might compensate (at least in part) for the poor adherence, provided that the mean daily dose of rhGH is high enough. In agreement with our findings, several studies have shown that for an rhGH dose of 0.03 mg/kg.d, three injections a week were as effective as six or seven a week (26, 27). However, another study concluded that daily injections were superior (28).

In agreement with the above mentioned hypotheses, we observed a non-significant trend toward a greater height increase for an rhGH dose<0.03 mg/kg.d vs.>0.03 mg/kg.d among fairly adherent patients (>5 injections/week) in the 12-month model (p = 0.07); This apparently paradoxical results suggests that the two categories of fairly adherent patients differed in their sensitivity to rhGH. The category with a dose<0.03 mg/kg.d was rhGH-sensitive and thus showed an adequate growth response to low-dose treatment. Accordingly, the growth response to treatment is usually good in rhGH-sensitive patients, such as those with profound GHD (25). In contrast, the category with a dose>0.03 mg/kg.d was probably rhGH-resistant: despite the administration of higher rhGH doses, the growth response to treatment may be smaller (25).

Taking into account a treatment period of 3 months when measuring the mean daily rhGH dose and the mean number of injections per week, our results showed that the yearly height gain SDS was not influenced by the variation in these two variables: short rhGH “treatment holidays” in treatment do not influence the yearly height gain SDS, provided that the variation does not go on for too long. In practical terms, this suggests that the treatment could indeed be stopped for short periods (one or two weeks of holidays for instance, but not more than 3 months) without a discernible effect on the yearly height gain. Of course, this finding reflected a flexible practice that might be country-specific. The difference with the one-year model was explained by the fact that the children in each dose/injection group were not the same in the 3-month model and the 12-month model. Thus, the high dose/fair adherence group in the 3-month model (45% of the 2741 patient trimesters) corresponded to patients who remained in this group (and who were found in the same group in the 12-month model) but also contained a number of patients who were in this group for one or more 3-month periods (though not permanently). Since the high dose/fair adherence group in the 12-month model corresponded to only 27% of the 685.2 patient years, this suggests that it was difficult for the children to be fully adherent for long periods. Accordingly, we observed a decrease in adherence with treatment duration. In other studies, good adherence was defined as performing>80% of the injections (16–18), and the proportion of patients with good adherence was 60 to 80% during the first few years of treatment (16). Although we used a different definition of adherence (see *Materials and Methods*), our adherence rate of 64% for the first year is at the lower end of the literature values reported in studies from various countries (29).

The present study had a number of strengths. Firstly, we recorded the true daily GH dose and the true number of injections per week; this enabled us to study adherence in periods rather than for individuals. Despite differences with the typically applied multiple regression models, our linear mixed models evidenced several conventional factors known to be associated with height SDS: the cause of the short stature, the midparental height, puberty, and the treatment duration (i.e. a waning treatment effect) (1–4).

Our study also had several limitations. Firstly, the number of participating subjects (n = 220) was small and the treatment duration (mean: 3.2 ± 1.1 years; range: 1.8 years to 5 years) was short; this may have affected the study’s power. Secondly, some treatment categories (rhGH dose >0.03 mg/kg.d with 3-4 injections/week and rhGH dose >0.03 mg/kg.d in<3 injections/week) corresponded to a small percentage of the patients’ treatment periods, and this might have weakened the study’s statistical power. However, pooling these categories gave the same findings. Secondly, and even though we were able to report the true main daily dose and the true number of injections per week, our interpretation of the results relied on a number of assumptions; hence, only randomized studies could demonstrate effects of the daily dose of rhGH and the number of injections on the height gain. Lastly, the study results reflected our flexible practice of increasing the prescribed GH dose in some patients to give a true GH dose of >0.03 mg/kg.d, despite a low number of weekly injections. This practice might be specific to France, and so our results cannot necessarily be generalized to other countries.

In conclusion, our study showed that poor adherence over one-year periods had an impact on the yearly height gain when the resulting daily rhGH dose was low. However, poor adherence for short periods (e.g. treatment cessation during a holiday) had no discernible effect on the yearly height gain.

## Data Availability Statement

The raw data supporting the conclusions of this article will be made available by the authors, without undue reservation.

## Ethics Statement

The studies involving human participants were reviewed and approved by French National Consultative Committee on Information Processing in Medical Research (Comité Consultatif sur le Traitement de l’Information en Matiere de Recherche dans le domaine de la Santé, Paris, France). Written informed consent to participate in this study was provided by the participants’ legal guardian/next of kin.

## Author Contributions

RC, BC, MT, MN, VB, and J-FH contributed to the conception and design of the study. J-FH and RC performed the statistical analysis. RC, MT, VB, and J-FH wrote the first draft of the manuscript. MN and BC revised the first draft of the manuscript. All authors approved the submitted version.

## Funding

The present analysis was funded by Merck Serono SAS, Lyon, France, an affiliate of Merck KGaA. Medical writing assistance was provided by David Fraser (Biotech Communication SARL, Ploudalmézeau, France) and funded by Merck Serono SAS, Lyon France, an affiliate of Merck KGaA. The Easypod™ Connect study was funded by Merck KGaA.

## Author Disclaimer

The views and opinions described in this publication do not necessarily reflect those of the grantor.

## Conflict of Interest

RC has received consulting fees, honoraria for lectures and/or research funding from Merck, Ipsen, Novo, Lilly, and Pfizer. MN has received consulting fees, honoraria for lectures and/or research funding from Merck, Ipsen, Novo, Lilly, and Pfizer. MT has received consulting fees, honoraria for lectures and/or research funding from Merck, Ipsen, Novo, and Pfizer. BC has received consulting fees, honoraria for lectures and/or research funding from Merck, Ipsen, Novo, Lilly, and Pfizer.

VB is an employee of Merck Serono SAS, an affiliate of Merck KGaA.

The remaining author declares that the research was conducted in the absence of any commercial or financial relationships that could be construed as a potential conflict of interest.

RC, MN, MT, and BC are pediatric endocrinologists who care for children of short stature, perform diagnostic work up for these patients, and initiate and monitor growth hormone treatment when appropriate. Neither they nor their spouses and children are employed by Merck Serono SAS or any other company that manufactures and markets growth hormone. J-FH is an academic statistician with no role in the care of children of short stature.

Merck Serono SAS provided the data to be analyzed but had no role in the choice of the statistical model, the statistical analyses, and the interpretation and discussion of the results. Merck Serono SAS had the right to comment on the draft manuscript but the authors retained the right to accept or reject comments.

## Publisher’s Note

All claims expressed in this article are solely those of the authors and do not necessarily represent those of their affiliated organizations, or those of the publisher, the editors and the reviewers. Any product that may be evaluated in this article, or claim that may be made by its manufacturer, is not guaranteed or endorsed by the publisher.
